# Automated breast ultrasound: are we ready to put it into practice in Brazil?

**DOI:** 10.1590/0100-3984.2020.53.5e2

**Published:** 2020

**Authors:** Linei Urban

**Affiliations:** 1 Clínica DAPI - Diagnóstico Avançado por Imagem, Curitiba, PR, Brazil. Email: lineiurban@hotmail.com.

Automated breast ultrasound (ABUS) is an ultrasound technique in which the entire breast volume is scanned with near-isotropic voxels, allowing image reconstruction in all planes. It was developed in the 1970s but was not widely accepted at the time because of the use of low-frequency transducers (4-7 MHz), which resulted in poor-quality images. However, with the increasing importance of ultrasonography in breast cancer screening, the interest in examination automation resurfaced. Currently, the systems use large (15-17 cm) transducers with a high frequency (10-14 MHz), coupled with a mechanical arm that slides over the breast, allowing a complete and good quality reading in approximately 15 min^(1,2)^.

The main advantage of ABUS in relation to manual or hand-held ultrasonography (HHUS) is that the standardized acquisition of images does not depend on the doctor, allowing greater reproducibility and access for all images retrospectively. With this technique, the image acquisition time is free from interpretation, as in mammography, tomosynthesis, and magnetic resonance imaging. Images are acquired by a trained technician or technologist, following a standardized protocol, and interpreted by a radiologist at a dedicated workstation, with a mean reading time of 3-10 min reported in the literature^(2,3)^.

Its clinical use was initially focused on the screening scenario, and studies were aimed at evaluating the technique in women with dense breasts. Although the evidence for long-term benefits is limited, screening with ABUS has demonstrated a high sensitivity for cancer detection, similar to HHUS. Several studies showed that cancer detection rates increased from 1.9 to 7.7 cases per 1,000 women. The sensitivity increased from 21.6% to 41.0%, but specificity varied. Recall and biopsy rates increased, while the positive predictive value 3 (PPV3) decreased from 4.2% to 15.8%^(1-4)^. A large ABUS study, called SomoInsight^(4)^, additionally detected 1.9 cancer cases per 1,000 women, similar to the randomized J-START study^(5)^ but with lower results than the ACRIN 6666^(6)^. Most tumors were invasive (93.3%), with a mean size of 12.9 mm and negative axillary lymph nodes (92.6%)^(4)^. Although the indications of ABUS for evaluating symptomatic patients, as in cases of papillary flow or palpable lesions, have been well studied, they remain uncertain^(1-3)^.

As with any technique, ABUS has some limitations. First, it is difficult for positioning in certain breast types, such as those with a large volume or implants. Second, it cannot evaluate some regions, such as the axillary region, or evaluate some regions with great difficulty, such as the retroareolar region. Third, the interpretation of subtle signs of malignancy may be difficult because of some artifacts that are exclusive to this technique. Therefore, trained and experienced physicians, technicians, and technologists play an important role in obtaining high-quality images, resulting in a qualified interpretation^(1-3,7)^. This is what Calas et al.^(8)^ demonstrated in the article published in this issue of Radiologia Brasileira. Only 1.1% of cases showed an unacceptable interpretation in the study. Further, in 19.5% of cases, the technician reported some difficulties, such as a rigid, large, small, or flaccid breast, high sternum, or difficult anatomy. In 6.8% of cases, the evaluating physician reported some limitations, such as the lack of compression, incomplete evaluation of the mammary region, and artifacts^(8)^.

Taken together, can we use ABUS in clinical practice in Brazil? The answer is complicated because the implementation of ABUS requires an important change in the way the examination is conducted, i.e., a change in the role played by the doctor during the examination. In Brazil, unlike other countries, such as the United States, ultrasound examinations are currently performed and interpreted by physicians, and an objective of automation is that examinations no longer depend on the physician and are performed by trained technicians or technologists. In this context, the radiologist is responsible for examination supervision, ensuring its correct execution within the appropriate technical parameters, interpreting its findings, and providing a medical report, as in mammography and magnetic resonance imaging. The physician is legally responsible for the examination. However, the question is whether or not there are qualified professionals to perform it since many variables must be controlled during the examination, such as adjusting the depth, focus, and gain, which needs to be optimized individually, and recognizing artifacts in image acquisition and when and how to resolve them. Another question is whether or not the patients will consent to undergoing the examination without the presence of a physician since they are used to doing it with a physician. These limitations may increase the false-positive test results and screening recall rates. Another factor is that the cost of this high-tech equipment is significantly higher compared to traditional devices as well as the final examination fee, which should include the technician or technologist, in addition to the physician, considering that ultrasonography is an examination with the highest cost gap in radiology^(3,7)^.

To summarize, ABUS is a new technique under development, which has the potential to overcome some limitations of conventional ultrasound, such as image acquisition standardization and examination reproducibility. However, further studies and a broad discussion are required to define issues related to workflow and cost-effectiveness as well as its role in complementary screening in Brazil.
